# A detailed investigation of anxiety disorders in children of clinically anxious parents: a population‐based study

**DOI:** 10.1111/jcpp.70085

**Published:** 2025-12-04

**Authors:** Sigrid Elfström, Susanne Wicks, Christina Dalman, Johan Åhlén

**Affiliations:** ^1^ Department of Global Public Health Karolinska Institutet Stockholm Sweden; ^2^ Center for Epidemiology and Community Medicine Region Stockholm Stockholm Sweden

**Keywords:** Anxiety disorders, familial risk, offspring, cohort study, population‐based study

## Abstract

**Background:**

We assessed the risk of anxiety disorders in children of clinically anxious parents, focusing on the influence of parent and child sex, parental care level, depressive comorbidity, and anxiety subtype, while controlling for socioeconomic factors and other parental psychiatric conditions.

**Methods:**

We conducted a population‐based study utilizing comprehensive healthcare data. A cohort of children (*N* = 516,134), born in 1998–2015 and residing in Stockholm, Sweden, was followed until they were diagnosed with anxiety, moved, or turned 18. The primary and secondary exposures were parental specified and unspecified anxiety diagnoses, respectively. The outcome was child specified anxiety diagnosis. Associations were estimated using hazard ratios (HRs) with 95% confidence intervals (CIs).

**Results:**

Among exposed children, 4.3% were diagnosed with specified anxiety disorders, compared to 3.0% of unexposed (HR: 1.44, 95% CI: 1.38, 1.51). Adjustment for socioeconomic factors and other parental psychiatric disorders attenuated the risk (HR: 1.27, 95% CI: 1.21, 1.34). The risk was higher when parental anxiety was recorded in specialized psychiatric care (HR: 1.69, 95% CI: 1.60, 1.79) than in primary care (HR: 1.24, 95% CI: 1.17, 1.32). Maternal anxiety was linked to a higher risk (HR: 1.49, 95% CI: 1.41, 1.56) than paternal (HR: 1.31, 95% CI: 1.21, 1.42). Children were most likely to develop the same anxiety disorder as their parents, in cases of social anxiety, specific phobia, and panic disorder. Parental unspecified anxiety diagnoses were not associated with an increase in risk (HR: 1.02, 95% CI: 0.98, 1.07).

**Conclusions:**

Parental specified anxiety modestly increased the risk of child anxiety disorders. While the overall risk was lower than previously reported, it varied across diagnosis types and care levels.

## Introduction

It is well documented that anxiety disorders run in families (Lawrence, Murayama, & Creswell, 2019; Micco et al., 2009; Uher et al., 2023). Traditional familial high‐risk studies often use structured diagnostic interviews to assess psychiatric disorders in small parent–child samples. As pointed out by Uher et al. (2023), this method ensures strong psychometric validity but poses a high risk of selection bias. It is therefore not surprising that the estimated risk for anxiety disorder in children of anxious parents has varied considerably across studies (Lawrence et al., 2019; Micco et al., 2009), with relative risk estimates ranging from below two to nearly eight. However, during the past two decades, advancements in the knowledge about family aggregation of anxiety disorders have been made, as several studies based on Nordic health‐care register data have been published (Helenius, Munk‐Jørgensen, & Steinhausen, 2014; Hyland, Shevlin, Elklit, Christoffersen, & Murphy, 2016; Kendler, Abrahamsson, Ohlsson, & Sundquist, 2022; Kendler, Abrahamsson, Ohlsson, Sundquist, & Sundquist, 2023; Li, Sundquist, & Sundquist, 2008; Steinhausen, Jakobsen, Meyer, Jørgensen, & Lieb, 2016; Sydsjö, Agnafors, Bladh, & Josefsson, 2018). Studies using high‐quality registers offer important advantages in terms of population‐based, representative samples. According to the results of several register‐based studies, the transmission of anxiety is higher from mothers than from fathers (Helenius et al., 2014; Hyland et al., 2016; Li et al., 2008), while the degree of parental transmission has been observed to be the same for both male and female offspring (Li et al., 2008). Further, the risk of anxiety disorders has been found to be higher if both parents are affected, compared to just one (Helenius et al., 2014; Li et al., 2008). In a recent meta‐analysis, data were synthesized from both traditional familial high‐risk studies and register‐based studies (Uher et al., 2023). The results showed that having a parent with an anxiety disorder is associated with an increased risk of developing an anxiety disorder, with a risk ratio of 2.2 and a lifetime risk of about 31%.

While register‐based research has advanced the understanding of the familial risk of anxiety disorders, there are still important knowledge gaps and methodological limitations in the literature. First, there is a need to distinguish the specific risk of having a parent with an anxiety disorder from the general risk of having a parent with any mental health condition. In existing register‐based studies, parental psychiatric comorbidity has generally not been comprehensively controlled for when estimating the risk for offspring anxiety, with only selected diagnoses considered (Hyland et al., 2016; Li et al., 2008) or none (Helenius et al., 2014; Kendler et al., 2023; Sydsjö et al., 2018). Second, several previous register‐based studies have applied broad diagnostic categories, clustering anxiety disorders together with obsessive‐compulsive disorder (OCD) and stress‐related disorders (Hyland et al., 2016; Li et al., 2008; Sydsjö et al., 2018). Third, no previous study on anxiety disorders, to our knowledge, has accounted for the chronological order of diagnoses between parent and child, which is key to assessing the risk of growing up with a clinically anxious parent.

Another knowledge gap concerns whether the severity of a parent's anxiety disorder affects the risk of offspring anxiety. Most previous register‐based studies (Helenius et al., 2014; Hyland et al., 2016; Li et al., 2008; Steinhausen et al., 2016; Sydsjö et al., 2018) rely solely on data from specialist psychiatric care, excluding primary care, which may have led to an underrepresentation of milder cases. To capture a more representative range of anxiety severity, there is a need to integrate data from all levels of care.

In this population‐based study, we aim to provide a robust estimate of the risk of developing anxiety disorders during childhood and adolescence associated with having a clinically anxious parent. Our analysis utilizes recent register data from all levels of health care, including primary, specialist, and inpatient services. To accurately assess the risk of growing up with an anxious parent, we considered only parental diagnoses that predated the child's diagnosis as exposure. The analyses accounted for potential confounding by socioeconomic status and other parental psychiatric conditions to isolate the specific effect of parental anxiety. Furthermore, to provide a more nuanced understanding of familial transmission of anxiety, we examined how risk is influenced by parent and child sex, parental level of care, depressive comorbidity, and type of anxiety diagnosis. These variables were selected based on prior findings (Helenius et al., 2014; Hyland et al., 2016; Kendler et al., 2022, 2023; Li et al., 2008; Steinhausen et al., 2016; Sydsjö et al., 2018) and clinical relevance, with the goal of identifying subgroups of children at particularly elevated risk. In line with previous research, we hypothesize stronger transmission from mothers than from fathers, and no difference in the level of risk between boys and girls. We also hypothesized that higher parental care levels (e.g., psychiatric specialist care) and comorbid depression might indicate greater clinical severity and thus greater familial risk. Additionally, we investigate whether specific developmental periods pose a heightened risk for children to develop an anxiety disorder.

## Methods

### Study design and data sources

We conducted a register‐based cohort study utilizing healthcare and administrative data linked via a unique individual identification number (Karolinska Institutet, 2024). The total population register was used to identify study participants, and the multi‐generation register to link parents and children. The Longitudinal Integration Database for Health Insurance and Labour Market Studies (LISA) was used to obtain socioeconomic data (e.g., parental income and education). To gather data on psychiatric diagnoses, we utilized records from primary, specialized, and inpatient care. Specifically, we used the Stockholm County VAL databases, comprising data from primary and specialized care, and the National Patient Register (NPR), comprising records of in‐ and specialized outpatient care in Sweden. To enhance coverage, we also included data from two Stockholm registers: PASTILL (child and adolescent psychiatry) and PVS (adult psychiatry). For a more detailed description of the component registers, see Ekholm et al. (2005), Idring et al. (2012), and Sellgren, Landén, Lichtenstein, Hultman, and Långström (2011).

Studies have shown that ICD diagnostic codes for several psychiatric disorders in the Swedish National Patient Register (NPR) have sufficient validity and reliability for research purposes (Ekholm et al., 2005; Idring et al., 2012; Sellgren et al., 2011). Concerning anxiety disorders, social anxiety disorder (SOC, Vilaplana‐Pérez et al., 2020) and hypochondriasis (Rautio et al., 2021) have been evaluated, both with acceptable diagnostic validity, with Positive Predictive Values of 0.8.

Ethical approval was obtained from the Swedish Ethical Review Authority (registration number 2010/1185–31/5). Informed consent was not collected, in accordance with the study's ethical permit and Swedish law (2003:460), which allows registry‐based research without consent if approved by an ethics review board and if there is minimal risk of discomfort or harm to participants. Before our team accessed the data, each participant's unique identification number was replaced with a serial number, ensuring that participants remained unidentifiable to us.

### Study population

The study population consisted of children born between 1998 and 2015, who were registered in Stockholm County for at least 4 years up to the year they turned 18. Children without identifiable parents (*n* = 182) and with adoptive guardians (*n* = 4,835) were excluded, leaving 516,134 children included in the final cohort. The children were followed until they were diagnosed with an anxiety disorder, moved from Stockholm, reached the end of the year they turned 18, or died, whichever occurred first. The follow‐up period spanned from January 1, 1998 to December 31, 2022, during which we obtained data on the children's anxiety diagnoses from healthcare registers.

We included only children in Stockholm due to the superior coverage of local psychiatric care registers compared to the national register.

### Definition of anxiety disorders

In Swedish healthcare registers, diagnoses are primarily recorded using the Statistical Classification of Diseases and Related Health Problems, 10th Revision (ICD‐10; World Health Organization, 2004). However, in PASTILL, the Stockholm register of specialized child psychiatry, diagnostic codes from the Diagnostic and Statistical Manual of Mental Disorders, Fourth Edition (DSM‐IV; American Psychiatric Association, 1994) are used. Table S1 lists the ICD‐10 and DSM‐IV codes categorized as anxiety disorders within this study. In our analysis, we distinguish between specified and unspecified anxiety diagnoses. This distinction is important because unspecified ICD codes are assigned when patients either do not meet the full criteria for specific anxiety disorders or the clinician lacks sufficient information for a precise diagnosis. Hence, including these unspecified codes alongside well‐defined anxiety disorder codes could compromise diagnostic validity.

In line with the ICD‐11 (World Health Organization, 2019) and DSM‐5 (American Psychiatric Association, 2013) we classify the following diagnoses as specified anxiety disorders: Agoraphobia, Social Anxiety Disorder, Specific Phobia, Panic Disorder, Generalized Anxiety Disorder, Illness Anxiety Disorder, Separation Anxiety Disorder, and Selective Mutism. For unspecified anxiety disorders, we categorized diagnostic codes such as ICD‐10 codes for Anxiety Disorder, Unspecified (F41.9), and Other Specified Anxiety Disorders (F41.8).

### Exposure variables

Parental specified anxiety disorder was the primary exposure; unspecified diagnosis served as secondary. To qualify as an exposure, the parental diagnosis had to be recorded before the child's anxiety diagnosis and registered any time from the year before the child's birth to the year they turned 18. Children were categorized as exposed to parental specified anxiety if they had parents diagnosed with either (1) only specified anxiety disorder(s) or (2) both specified and unspecified anxiety disorders. Conversely, children were categorized as exposed to unspecified anxiety only if their parents were diagnosed exclusively with unspecified anxiety.

To further investigate the impact of exposure to parental specified anxiety, we conducted multiple subgroup analyses. In Stockholm County, primary care is responsible for the initial assessment and treatment of mild to moderate mental health problems, including anxiety disorders. Patients with more severe psychiatric conditions (e.g., with severe impairment, psychiatric comorbidities, or an insufficient response to adequate treatment in primary care) are referred to specialized psychiatric services (Lindgren, 2016; VISS, 2022). To assess how the severity of parental anxiety influences the risk, we analyzed data separately based on whether parental specified anxiety diagnoses were recorded in specialized psychiatric care or other healthcare settings. As approximately 90% of the specified anxiety diagnoses from other healthcare settings were registered in primary care, we labeled the categories primary care and specialist psychiatric care. Furthermore, separate analyses were conducted to assess the risk associated with having a parent diagnosed with (1) specified anxiety and depression, and (2) specified anxiety with no occurrence of depression. To qualify as exposure, parental anxiety and depression diagnoses did not need to be registered during the same period; however, both had to be recorded before the child's anxiety diagnosis. Additionally, we evaluated the risks linked to exposure to specific parental anxiety disorders, including agoraphobia, SOC, specific phobia (SP), panic disorder (PD), generalized anxiety disorder (GAD), and illness anxiety disorder.

### Covariates

Parental income, educational level, country of birth, social welfare receipt, employment status, and parental cohabitation were considered potential confounders, as we aimed to ensure that adverse social conditions did not account for the association between parental and child anxiety. Information on the confounders was collected for the child's birth year. Parental income was estimated by collecting data on annual disposable family income from the LISA register. The variable is estimated by Statistics Sweden, based on annual total family income (including wages, welfare benefits, and pensions) and weighted with the total number of children and their ages. The annual disposable income of the parents was grouped into quintiles, based on the total population that year. If a child's parents fell into different income groups, the higher income of the two was recorded. Parental education level was stated as the highest level reached by any of the child's parents, categorized into compulsory (0–9 years), secondary (10–12 years), and university (≥13 years). Parental country of birth was defined as: all known parents born outside of Sweden, one parent born outside of and one in Sweden, and all known parents born in Sweden. Receipt of social welfare benefits was defined as at least one parent receiving municipal financial assistance. Employment status was defined as having at least one parent or no parent with gainful employment. Parental cohabitation status was defined as the child being registered at the same address as both parents or not.

Additionally, other parental psychiatric disorders, registered from 1 year before the child was born until the year the child turned 18, were seen as possible confounders. The psychiatric diagnosis and their ICD codes analyzed as possible confounders were substance use disorder (F10–F16, F18–F19), psychotic disorders (F20–F29), bipolar disorder (F30–F31), depressive episode or recurrent depressive disorder (F32‐F33), other mood disorders (F34, F38–F39), obsessive‐compulsive disorder (F42), reaction to severe stress and adjustment disorders (F43), eating disorders (F50), personality disorders (F60), ADHD (F90), and pervasive developmental disorders (F84).

### Outcome

The primary outcome was the child's first occurrence of a specified anxiety disorder. We also analyzed individual anxiety disorder subtypes in the child as outcomes, including SOC, SP, PD, GAD, and separation anxiety disorder. Due to insufficient power, selective mutism, agoraphobia, and illness anxiety disorder were excluded as separate outcomes.

### Statistical methods

Children exposed to parental anxiety disorders were compared to unexposed children. We applied Cox regression with mixed effects to estimate hazard ratios (HR) with 95% confidence intervals (CI). Cox models are well suited for our study design, as they accommodate differing follow‐up times and allow individuals to enter and exit the risk set at different time points. The underlying timescale for the survival analysis was age, meaning that each participant's time‐to‐event was estimated from the age at entry to age at censoring. Using age as the timescale enables analysis of age‐specific risk and is recommended for cohort studies to reduce bias (Thiébaut & Bénichou, 2004).

To systematically address the study aim, we explored three models. The first provided a crude estimate, adjusted only for birth year and sibling clustering. Adjustment for birth year was included to account for potential time‐related variations in diagnostic practices, register coverage, and differences in follow‐up duration across birth cohorts. We applied sibling clustering adjustment (using family as a random effect), recognizing that children exposed to the same parent's anxiety should not be treated as independent observations. The second model added an adjustment for socioeconomic factors and child sex. In the third and fully adjusted model, we additionally accounted for other parental psychiatric disorders. This stepwise approach allowed us to judge the extent to which different groups of confounders contribute to explaining the observed association, and at the same time enabled us to present a final model adjusted for a comprehensive number of confounders, providing a conservative estimate that accounts for a broad range of potential background influences. For models 2 and 3, individuals with missing data on any confounders were excluded from the analyses (*n* = 59,135, 11.5%).

Furthermore, we estimated separate risks (HR) based on parent and child sex, level of care, and parental depressive comorbidity. These estimates were statistically compared within the crude model (model 1), with a *p*‐value below 0.05 considered statistically significant.

We used the crude model (model 1) to explore separate Cox regressions for individual child anxiety disorders (e.g., PD). In these analyses, all parental‐specified anxiety disorders were included as exposures to evaluate the specific contribution of each disorder while controlling for the others. We explored whether there may be sensitive developmental periods during which children may be at heightened risk for developing an anxiety disorder by plotting the age‐specific incidence rates and risk ratios of childhood anxiety by exposure status. These were presented in descriptive plots, intended to illustrate potential patterns over age, without formal statistical testing of group differences or slopes.

All statistical analyses were performed in the R software (R Core Team, 2022) using the “Coxme” package.

## Results

Demographic characteristics of the children (*N* = 516,134) and clinical characteristics of their parents can be seen in Table 1. Regarding the main exposure, 11.8% of the cohort children had at least one parent diagnosed with a specified anxiety disorder during the study follow‐up period. Specifically, 8.4% of the children were exposed to only maternal, 4.0% to only paternal, and 0.6% to both maternal and paternal specified anxiety disorders. Regarding the secondary exposure, 18.3% of children were exposed only to unspecified parental anxiety. Table S2 presents the number of children exposed to parental anxiety disorders, categorized by exposure to each specific anxiety subtype.

**Table 1:** Baseline demographics of the full cohort of children (*N* = 516,134) divided by exposed children (*n* = 61,144) and unexposed children (*n* = 454,990)

Characteristics	All children *N* (%)	Exposed *N* (%)	Unexposed *N* (%)
Birth year			
Born 1998–2003	152,312 (29.5%)	16, 533 (27.0%)	135,779 (29.8%)
Born 2004–2009	181,034 (35.1%)	23, 079 (37.7%)	157, 955 (34.7%)
Born 2010–2015	182, 788 (35.4%)	21,532 (35.2%)	161,256 (35.4%)
Parent educational level			
≤9 years (Primary education/Compulsory school)	24,241 (4.7%)	4,429 (7.2%)	19,812 (4.4%)
12 years (Upper secondary education/high school)	143,081 (27.7%)	22,673 (37.1%)	120,408 (26.5%)
>12 years (University/collage)	296,193 (57.4%)	31,567 (51.6%)	264,626 (58.2%)
Parental education level missing	52,619 (10.2%)	2,475 (4.1%)	50,144 (11.0%)
Parent county of birth			
All known parents born outside Sweden	141, 321 (27.4%)	11,659 (19.1%)	129, 662 (28,5%)
One parent born outside and one inside Sweden	84,740 (16.4%)	11,498 (18.8%)	73,242 (16.1%)
All known parents born in Sweden	290, 073 (56.2%)	37, 987 (62.1%)	252, 086 (55.4%)
Parent Birth Country Missing	0 (0%)	0 (0%)	0 (0%)
Parental cohabitation status			
Cohabiting parents	424, 642 (82.3%)	53, 082 (86.8%)	371,560 (81.66%)
Parents not living together	35,165 (6.81%)	5, 319 (8.7%)	29, 846 (6.6%)
Parental cohabitation status missing	56, 327 (10.9%)	2, 743 (4.49%)	53, 584 (10.4%)
Parental anxiety and other psychiatric disorders			
Exposed to maternal specified anxiety	43,516 (8.43%)	43,516 (71.2%)	
Exposed to paternal specified anxiety	20, 484 (3.97%)	20,484 (33.5%)	
Exposed to maternal unspecified anxiety^a^	101,879 (19.7%)	28,113 (46.0%)	73,766 (16.2%)
Exposed to paternal unspecified anxiety^b^	49,851 (9.7%)	11,751 (19.2%)	38,100 (8.4%)
Exposed to maternal other psychiatric diagnosis	198, 247 (38.4%)	43,697 (71.5%)	154,550 (34.0%)
Exposed to maternal other psychiatric diagnosis	198, 247 (38.4%)	43,697 (71.5%)	154,550 (34.0%)
Exposed to paternal other psychiatric diagnosis	112, 188 (21.7%)	26,777 (43.8%)	85,411 (18.8%)

^a^ Children not exposed to maternal specified anxiety but exclusively to maternal unspecified anxiety.

^b^ Children not exposed to paternal specified anxiety but exclusively to paternal unspecified anxiety.

For the main outcome, 3.6% of children in the cohort were diagnosed with a specified anxiety disorder during the follow‐up period, with a higher incidence among girls (4.4%) than boys (1.9%). Among children not exposed to parental specified anxiety, 3.1% developed a specified anxiety disorder, compared to 4.6% among exposed children. The incidence of anxiety disorders within the cohort is detailed in Table 2.

**Table 2:** Number and percentages of children (*N* = 516,134) with a registered anxiety disorder divided by girls (*n* = 250,509) and boy (*n* = 265,625), and exposed (61,144) and unexposed (454,990)

Child anxiety disorder	All children, *n* (%)	Girls, *n* (%)	Boys, *n* (%)	Exposed, *n* (%)	Unexposed, *n* (%)
Child any specified AD	16,100 (3.1)	11,029 (4.4)	5,071 (1.9)	2,651 (4.3)	13,449 (3.0)
Child AG	640 (0.1)	515 (0.2)	125 (0.0)	101 (0.2)	539 (0.1)
Child Soc	4,819 (0.9)	3,751 (1.5)	1,068 (0.4)	797 (1.3)	4,022 (0.9)
Child SP	3,708 (0.7)	2,325 (0.9)	1,383 (0.5)	598 (1.0)	3,110 (0.7)
Child PD	3,460 (0.7)	2,688 (1.1)	772 (0.3)	598 (1.0)	2,862 (0.6)
Child GAD	3,498 (0.7)	2,467 (1.0)	1,031 (0.4)	609 (1.0)	2,889 (0.6)
Child IAD	161 (0.0)	95 (0.0)	66 (0.0)	22 (0.0)	139 (0.0)
Child Separation Anxiety	1,006 (0.2)	601 (0.2)	405 (0.2)	109 (0.2)	897 (0.2)
Child Selective Mutism	1925 (0.4)	1,071 (0.4)	854 (0.3)	380 (0.6)	1,545 (0.3)

AD, anxiety disorder; AG, agoraphobia; GAD, generalized anxiety disorder; IDA, illness anxiety disorder; PD, panic disorder; SEP, separation anxiety disorder; Soc, social anxiety disorder; SP, specific phobia.

Table 3 presents hazard ratios (HRs) and confidence intervals (CIs) for children's risk of specified anxiety disorders based on parental anxiety exposure, divided across three models: crude, adjusted for sex and socioeconomic factors, and fully adjusted for sex, socioeconomic factors, and parental psychiatric comorbidity. Children with at least one parent diagnosed with a specified anxiety disorder had an increased risk for being diagnosed with a specified anxiety disorder themselves. In the crude model, the HR was 1.44 (95% CI: 1.38, 1.51), which remained largely unchanged after adjusting for socioeconomic variables, HR = 1. 42 (95% CI: 1.35, 1.48). Further adjustment for both socioeconomic factors and other parental psychiatric disorders reduced the HR to 1.27 (95% CI: 1.21, 1.34).

**Table 3:** Hazard ratios (HR) and 95% confidence interval of child‐specified anxiety disorder for children exposed to parental‐specified anxiety disorder compared to unexposed children

Exposure	Outcome	Model 1	Model 2	Model 3
		HR (95% CI)	HR (95% CI)	HR (95% CI)
Any parent with specified anxiety disorder	All children	1.44 (1.38,1.51)	1.42 (1.35, 1.48)	1.27 (1.21,1.34)
Any parent with specified anxiety disorder	Boys	1.51 (1.40, 1.63)	1.47 (1.36, 1.59)	1.32 (1.22,1.43)
Any parent with specified anxiety disorder	Girls	1.44 (1.37, 1.52)	1.39 (1.32, 1.47)	1.25 (1.18, 1.33)
Any parent with specified anxiety disorder in primary care	All children	1.24 (1.17,1.32)	1.24 (1.16, 1.32)	1.13 (1.06,1.21)
Any parent with specified anxiety disorder in psychiatric care	All children	1.69 (1.60,1.79)	1.63 (1.53, 1.73)	1.45 (1.36,1.54)
Only mother with specified anxiety disorder	All children	1.49 (1.41, 1.56)	1.46 (1.39, 1.54)	1.32 (1.24, 1.39)
Only father with specified anxiety disorder	All children	1.31 (1.21, 1.42)	1.28 (1.18, 1.39)	1.16 (1.07, 1.26)
Mother and father with specified anxiety disorder	All children	1.68 (1.42, 1.99)	1.63 (1.37, 1.94)	1.44 (1.21, 1.71)
Any parent with unspecified anxiety disorder	All children	1.02 (0.98,1.07)	1.02 (0.97, 1.06)	0.91 (0.87, 0.96)
Any parent with specified anxiety disorder without depression	All children	1.40 (1.31, 1.50)	1.39 (1.29, 1.49)	
Any parent with specified anxiety disorder and depression	All children	1.47 (1.39, 1.55)	1.43 (1.36, 1.52)	

In the crude model, exposed girls and boys had similar risks of developing a specified anxiety disorder, with no significant difference (HR = 1.44 for girls vs. HR = 1.51 for boys, *p* = 0.317).

The HR for children with only a mother diagnosed with specified anxiety was higher compared to those with only a diagnosed father (HR = 1.49 vs. HR = 1.31, *p* = .007). Further, the risk for children with both parents diagnosed with specified anxiety disorders was higher than for children with only a father diagnosed (HR = 1.68 vs. 1.31, *p* = .009) but not for children with only a mother diagnosed (HR = 1.68 vs. 1.49, *p* = .170).

Children with a parent diagnosed with a specified anxiety disorder in specialized psychiatric care had a statistically higher risk of being diagnosed themselves compared to children with a parent diagnosed in primary care (HR = 1.69 vs. 1.24, *p* < .001).

Children exposed to both parental specified anxiety and depression had a similar risk as those exposed to parental specified anxiety alone (HR = 1.47 vs. HR = 1.40, *p* = 0.271). Exposure to parental diagnoses of unspecified anxiety disorder showed no increased risk (HR = 1.02 (95% CI: 0.98, 1.07)).

Overall, the results in Table 3 show that the HRs were minimally attenuated due to child sex and socioeconomic factors. In contrast, the HRs were substantially attenuated by other parental psychiatric conditions, indicating that part of the effect could be attributed to the broader influence of parental psychopathology.

In Table 4, the associations between specific parental anxiety disorders and the risk of corresponding anxiety disorders in children are presented. Parental social anxiety disorder (SOC) was strongly associated with an increased risk of SOC in children, with an HR of 1.96 (95% CI: 1.67, 2.31). Similarly, parental specific phobia (SP) was linked to a twofold increase in risk for SP in children (HR 2.00, 95% CI: 1.46, 2.75). Further, parental generalized anxiety disorder (GAD) was associated with an elevated risk of GAD in children (HR 1.59, 95% CI: 1.39, 1.82), and parental panic disorder (PD) with an elevated risk of PD in children (HR 1.56, 95% CI: 1.39, 1.75).

**Table 4:** Hazard ratios (HR) and 95% confidence interval of individual child‐specified anxiety disorders for children exposed to parental‐specified anxiety disorders compared to unexposed children (model 1)

	Parent SOC	Parent, GAD	Parent, PD	Parent, SP	Parent IAD	Parent, AG
	HR (95% CI)	HR (95% CI)	HR (95% CI)	HR (95% CI)	HR (95% CI)	HR (95% CI)
Child, any specified AD	**1.31 (1.19, 1.45)**	**1.27 (1.18, 1.36)**	**1.28 (1.21, 1.36)**	**1.45 (1.24, 1.70)**	1.15 (0.93, 1.44)	1.01 (0.88, 1.16)
Child, SOC	**1.96 (1.67, 2.31)**	**1.19 (1.05, 1.35)**	**1.27 (1.14, 1.41)**	1.34 (1.00, 1.81)	0.94 (0.60, 1.47)	0.91 (0.71, 1.17)
Child, GAD	1.14 (0.91, 1.42)	**1.59 (1.39, 1.82)**	**1.27 (1.13, 1.44)**	**1.65 (1.21, 2.25)**	**1.58 (1.06, 2.35)**	0.94 (0.70, 1.25)
Child, PD	1.12 (0.89, 1.40)	**1.27 (1.10, 1.47)**	**1.56 (1.39, 1.75)**	1.28 (0.89, 1.83)	1.11 (0.68, 1.80)	1.14 (0.87, 1.50)
Child, SP	1.05 (0.82, 1.35)	1.10 (0.93, 1.29)	1.09 (0.95, 1.26)	**2.00 (1.46, 2.75)**	1.30 (0.81, 2.09)	1.04 (0.74, 1.46)
Child, SEP	1.05 (0.79, 1.39)	**1.73 (1.46, 2.04)**	**1.38 (1.18, 1.61)**	1.48 (0.99, 2.22)	0.78 (0.40, 1.51)	1.03 (0.71, 1.48)

Gray‐shaded cells indicate the risk of developing the same subtype of anxiety as the parent.

AD, anxiety disorder; AG, agora phobia; GAD, generalized anxiety disorder; IDA, illness anxiety disorder; PD, panic disorder; SEP, separation anxiety disorder; Soc, social anxiety disorder; SP, specific phobia.

**Figure 1:**
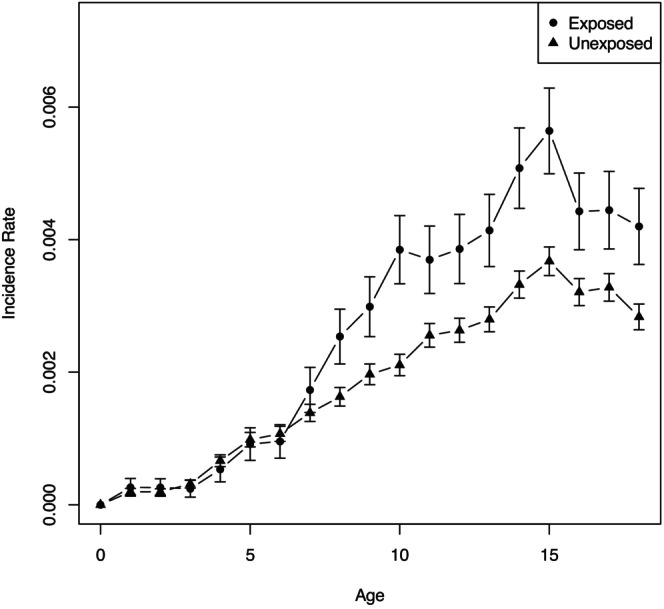
Age‐specific incidence rates and 95% confidence interval of childhood anxiety disorders divided by exposure status

**Figure 2:**
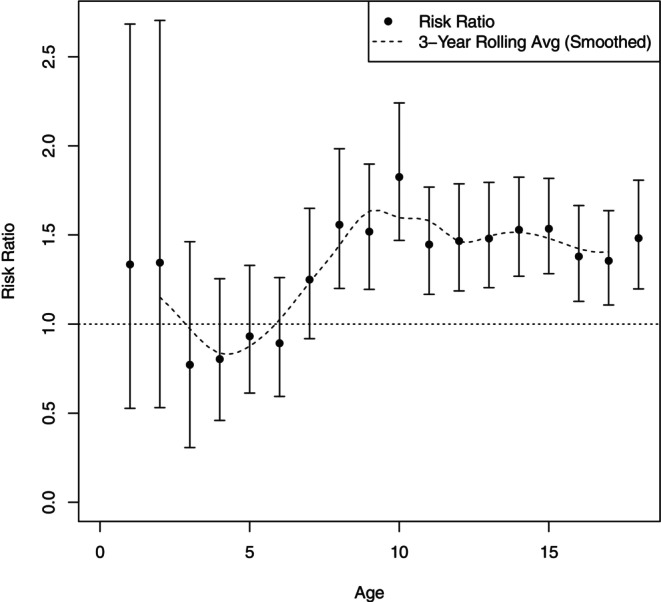
Age‐specific risk ratios, 95% confidence intervals, and 3‐year rolling averages of childhood anxiety disorders divided by exposure status

Figure 1 indicates that the incidence rate of childhood anxiety increases with age for both exposed and unexposed children. The curves largely follow the same overall pattern, but begin to diverge around age six, after which the incidence rate is consistently higher among children exposed to parental anxiety. The largest visible differences in slopes appear between ages 6–10 and 13–15, suggesting two potentially sensitive periods for children exposed to parental anxiety. In Figure 2, the highest risk ratio was observed between ages 9–10, highlighting this period as a possible sensitive age for children with anxious parents.

## Discussion

This study aimed to conduct a detailed examination of the risk of anxiety disorders in children of parents with anxiety disorders, considering the sex of the parent and child, level of care, parental depressive comorbidity and type of anxiety diagnosis. Overall, the results indicate a modest risk increase associated with exposure to parental specified anxiety disorder, attenuated after adjusting for other parental psychiatric conditions. Consistent with previous research (Helenius et al., 2014; Hyland et al., 2016; Li et al., 2008), exposure to maternal anxiety disorder was associated with a slightly higher risk than paternal anxiety, and the risk associated with being exposed to parental anxiety did not vary between girls and boys. The risk for children with both parents diagnosed with a specified anxiety disorder was higher than for those with only a father diagnosed, but not significantly higher than for those with only a mother diagnosed. Having a parent with an unspecified anxiety diagnosis was not associated with increased risk for a specified anxiety disorder in the child.

A key contribution of this study is the comparison of anxiety disorder risk across levels of care. Children of parents diagnosed in specialized psychiatric care had a higher risk than those with parental diagnoses in primary care, suggesting that children of parents with more severe anxiety face greater risk than those with milder anxiety.

In previous register‐based studies (Helenius et al., 2014; Hyland et al., 2016; Kendler et al., 2023; Li et al., 2008; Sydsjö et al., 2018; Uher et al., 2023), the risk for offspring anxiety disorders has been estimated to be higher than in the current study. This discrepancy is likely partly explained by the fact that many earlier studies relied solely on data from specialized psychiatric care, excluding primary care data. For example, Li et al. (2008) used only inpatient data, utilizing registers dating back to the 1930s. It is important to consider that patients treated within psychiatric care several decades ago may not be comparable to those receiving care today. By basing the analysis on newer register data covering all levels of care, as done in the current study, the results become more relevant for clinicians and researchers working to support affected families today.

The results from the current study reveal an interesting pattern: the elevated risk of pediatric anxiety disorders appears to partly be specifically linked to parental anxiety disorders, rather than any parental mental illness and seems in part unique to each anxiety disorder subtype. First, children whose parents were diagnosed with a specified anxiety disorder were found to have an elevated risk, whereas children whose parents were diagnosed with unspecified anxiety did not exhibit an increased risk of developing an anxiety disorder themselves. Further, the increased risk associated with being exposed to parental specified anxiety remains after controlling for other parental psychiatric conditions. Finally, children whose parents had social anxiety, specific phobia, or panic disorder were at the highest risk of developing the same anxiety condition as their parents. Twin studies have found genetic influence on child anxiety disorders (Cheesman, Rayner, & Eley, 2019; Knopik, Neiderhiser, DeFries, & Plomin, 2017), and the disorder‐specific risk could arise from specific genetic factors. One twin study found evidence of both common genetic influences across internalizing disorders and disorder‐specific genetic factors, suggesting that while a general genetic risk plays a more significant role in increasing the likelihood of developing anxiety, specific genetic components may contribute to the transmission of the same anxiety disorder (Lahey, Van Hulle, Singh, Waldman, & Rathouz, 2011). It is also possible that the disorder‐specific pattern observed in the current study is explained by environmental transmission. Although findings have not been consistent, some twin studies support shared environmental effects on child anxiety (Cheesman et al., 2019; Knopik et al., 2017). Further, studies with adoption and ‘children of twins’ designs support environmental transmission of anxiety from parent to child (Eley et al., 2015; Kendler et al., 2022).

A possible pathway for environmental transmission is model learning, as experimental studies have shown that children can acquire fear by observing their parents (de Rosnay, Cooper, Tsigaras, & Murray, 2006; Gerull & Rapee, 2002). This mechanism may be particularly relevant for explaining the disorder‐specific pattern observed in our study, whereby children appear likely to develop the same subtype of anxiety as their parents. Other dimensions of parenting, such as overprotection and family accommodation, have also been associated with increased anxiety in children (Lebowitz et al., 2013; Wood, McLeod, Sigman, Hwang, & Chu, 2003). If anxiety is partly transmitted from parent to child environmentally, the most involved parent likely has the greatest impact on the child's anxiety risk. A recent report indicates that Swedish women spend twice as much time caring for their children compared to men (Statistics Sweden, 2021). Notably, in line with previous findings, we found that exposure to maternal anxiety was linked to a higher risk of child anxiety compared to exposure to paternal anxiety. The heightened risk associated with maternal anxiety could be an indication of environmental transmission.

Anxiety is a typical response to life stressors and a normal part of human development (Gullone, 2000; Marks & Nesse, 1994), and a common symptom in patients across a wide range of psychiatric conditions (Astill Wright et al., 2023; Lau et al., 2020). Accordingly, many patients presenting with anxiety in healthcare settings do not exhibit the symptom profile characteristic of anxiety disorders. The finding that parental unspecified anxiety does not entail an elevated risk for child specified anxiety suggests that unspecified and specified anxiety codes capture different issues. Unspecified anxiety codes are likely used to document a variety of concerns that do not align with the clinical features of clearly defined anxiety disorders. This has methodological implications for future register‐based studies, indicating that specified anxiety codes should be analyzed separately from unspecified codes to ensure validity.

Anxiety disorders are the most prevalent psychiatric conditions among children (Polanczyk, Salum, Sugaya, Caye, & Rohde, 2015), yet effective prevention programs remain scarce (Lawrence, Rooke, & Creswell, 2017). A relatively new approach to anxiety prevention involves targeting families where parents have anxiety disorders (Havinga et al., 2021). The findings of this study hold important implications for researchers developing programs for families with anxious parents. As the results suggest a small increase in risk for children of parents diagnosed with anxiety in primary care and no increased risk associated with exposure to parental unspecified anxiety diagnosis, parental anxiety of lower severity may not, on its own, be the most useful indicator for identifying families who would benefit most from preventive interventions. Prevention efforts may be more effectively directed toward families where parental anxiety is more severe or co‐occurs with additional risk factors.

A major strength of this study is its large population cohort, encompassing all children aged 0–18 born in 1998–2015 and living in the Stockholm Region during the study follow‐up period. In Sweden, healthcare is free for children, and for adults patient fees are low and capped by a national high‐cost protection scheme, ensuring that healthcare is available to patients across diverse socioeconomic backgrounds. Healthcare registers therefore capture patients across demographic backgrounds. The use of high‐quality healthcare registers, covering all levels of care, adds robustness to our findings. The large sample size provided strong statistical power, allowing for a detailed examination of how anxiety severity and diagnosis type influence anxiety transmission from parent to child. Another key strength is our capacity to adjust for various socioeconomic factors and other parental psychiatric conditions.

One important limitation is that many individuals with psychiatric disorders never seek care and, therefore, do not have a recorded diagnosis. Those who have an anxiety disorder noted in healthcare registers may not be representative of the population of individuals with undiagnosed anxiety disorders, which could impact the generalizability of the findings. Further, the quality of research based on register data is entirely dependent on the validity of the registered diagnostic codes. The validity of a wide range of ICD diagnostic codes for psychiatric disorders in the Swedish National Patient Register (NPR) has been examined, with findings indicating sufficient validity and reliability for research purposes. However, only a limited subset of anxiety diagnoses has undergone specific validation. Another limitation is that socioeconomic status (SES) was measured only at birth, which does not capture potential changes in family SES over time or the cumulative impact of socioeconomic disadvantage throughout childhood.

Finally, a limitation of this study is the uncertainty regarding whether parents with anxiety disorders differ from those without in their likelihood of seeking care for their children's anxiety. If such differences exist, they could potentially skew the findings. Parents with anxiety might be more likely to seek care for their children's anxiety due to their heightened ability to recognize symptoms and greater awareness of treatment options. Additionally, it is possible that parents with specific subtypes of anxiety (e.g., social anxiety) are particularly attuned to detecting similar symptoms in their child. This tendency could result in an overestimation of the risk of children developing the same anxiety disorder as their parent. Conversely, anxious parents might perceive anxiety as normal, which could make them less likely to seek professional help for their child. This latter possibility could instead lead to an underestimation of the risk.

## Conclusion

This study provides evidence for a modestly increased risk of pediatric anxiety disorder associated with exposure to parental anxiety disorder. The findings highlight that the estimated risk varies based on the severity and the specific subtype of the parental anxiety diagnosis.

## Ethical considerations

Ethical approval was obtained from the Swedish Ethical Review Authority (registration number 2010/1185–31/5). Informed consent was not collected, in accordance with the study's ethical permit and Swedish law (2003:460), which allows registry‐based research without consent if approved by an ethics review board and if there is minimal risk of discomfort or harm to participants. Before our team accessed the data, each participant's unique identification number was replaced with a serial number, ensuring that participants remained unidentifiable to us.

**Key points**

**What's known?**

• Anxiety disorders are known to run in families. This population‐based cohort study, including 516,134 children, examined the risk of anxiety disorders in children of affected parents.

**What's new?**

• Higher risks were observed for children of parents diagnosed within specialized psychiatric care compared to primary care. Maternal anxiety posed a higher risk than paternal.
• In cases of social anxiety, specific phobia, and panic disorder, children were most likely to develop the same anxiety disorder as their parent.
• Parental anxiety was linked to a modestly increased risk of child anxiety, attenuated after adjusting for other parental psychiatric conditions.

**What's relevant?**

• Families where parents have more severe anxiety (i.e., parents treated in specialized care) should be prioritized for family and preventive interventions.

## Supporting information


**Table S1.** Diagnostic codes utilized to identify anxiety disorders in the current study, listed by classification system.
**Table S2.** Number and percentages of children (*N* = 516,134) exposed to specified parental anxiety disorders.

## Data Availability

Research data are not shared.
