# Supplementary material for: A detailed investigation of anxiety disorders in children of clinically anxious parents: a population‐based study
Source: J Child Psychol Psychiatry. 2025 Dec 4;67(6):919–28. doi: 10.1111/jcpp.70085 (PMC13170625; doi:10.1111/jcpp.70085)
Supplement: Supplementary file 1 — Table S1. Diagnostic codes utilized to identify anxiety disorders in the current study, listed by classification system. Table S2. Number and percentages of children (N = 516,134) exposed to specified parental anxiety disorders. [file JCPP-67-919-s001.docx]

**A Detailed Investigation of Anxiety Disorders in Children of Clinically Anxious Parents: A Population-Based Study**

**Supporting Information**

**Table S1**

*Diagnostic Codes Utilized to Identify Anxiety Disorders in the Current Study, Listed by Classification System*

|  | ICD-10 | DSM-4 |
| --- | --- | --- |
| **Specified Anxiety Disorders** |  |  |
| Agoraphobia | F40.0 | 300.22 |
| Social Anxiety Disorder | F40.1, F93.2 | 300.23, |
| Specific Phobia | F40.2, F40.8, F93.1 | 300.29 |
| Generalized Anxiety Disorder | F41.1 | 300.02 |
| Panic Disorder | F41.0 | 300.01, 300.21 |
| Illness Anxiety Disorder/ Hypochondriasis | F45.2 | 300.7 |
| Separation Anxiety | F93.0 | 309.21 |
| Selective Mutism | F94 | 313.23 |
| **Unspecified Anxiety** | F40.9, F41.2, F41.3, F41.8, F41.9, F41.8 | 300.00 |

*Notes:* ICD-10= International Classification of Diseases, 10th Revision, DSM-4: Diagnostic and Statistical Manual of Mental Disorders, Fourth Edition.

**Table S2**

*Number and percentages of children (N=516,134****)*** *exposed to specified parental anxiety disorders*

| Parent disorders | Child exposed to parental disorder | Child exposed to maternal disorder | Child exposed to paternal disorder |
| --- | --- | --- | --- |
| Agoraphobia | 5389 (1.1%) | 3711 (0.7%) | 1723 (0.3%) |
| Social Anxiety | 9929 (1.9%) | 6516 (1.3%) | 3543 (0.7%) |
| Specific Phobia | 3611 (0.7%) | 2849 (0.6%) | 771 (0.2%) |
| Panic Disorder | 35,329 (6.8%) | 24,225 (4.7%) | 12,128 (2.3%) |
| Generalized Anxiety Disorder | 25,028 (4.8%) | 18369 (3.6%) | 7095 (1.4%) |
| Illness Anxiety Disorder | 2073 (0.4%) | 1384 (0.3%) | 691 (0.1%) |
